# The cumulative risk of jail incarceration

**DOI:** 10.1073/pnas.2023429118

**Published:** 2021-04-12

**Authors:** Bruce Western, Jaclyn Davis, Flavien Ganter, Natalie Smith

**Affiliations:** ^a^Department of Sociology, Columbia University, New York, NY 10027;; ^b^Department of Political Science, Columbia University, New York, NY 10027;; ^c^Yale Law School, Yale University, New Haven, CT 06520

**Keywords:** incarceration, race, poverty

## Abstract

Research on incarceration has focused on prisons, but jail detention is far more common than imprisonment. Jails are local institutions that detain people before trial or incarcerate them for short sentences for low-level offenses. Research from the 1970s and 1980s viewed jails as “managing the rabble,” a small and deeply disadvantaged segment of urban populations that struggled with problems of addiction, mental illness, and homelessness. The 1990s and 2000s marked a period of mass criminalization in which new styles of policing and court processing produced large numbers of criminal cases for minor crimes, concentrated in low-income communities of color. In a period of widespread criminal justice contact for minor offenses, how common is jail incarceration for minority men, particularly in poor neighborhoods? We estimate cumulative risks of jail incarceration with an administrative data file that records all jail admissions and discharges in New York City from 2008 to 2017. Although New York has a low jail incarceration rate, we find that 26.8% of Black men and 16.2% of Latino men, in contrast to only 3% of White men, in New York have been jailed by age 38 y. We also find evidence of high rates of repeated incarceration among Black men and high incarceration risks in high-poverty neighborhoods. Despite the jail’s great reach in New York, we also find that the incarcerated population declined in the study period, producing a large reduction in the prevalence of jail incarceration for Black and Latino men.

## Significance

Research on incarceration has focused on prisons, but the scale of admissions to local jails is significantly higher. The average length of stay in jail is brief compared to imprisonment, but jail incarceration has been found to adversely affect court outcomes, earnings, and family life. Although New York has one of the lowest jail incarceration rates among large cities, over a quarter of Black men and a sixth of Latino men in the city have been jailed by age 38 y. Risks of jail incarceration are 40 to 50% higher in poor neighborhoods.

Research on mass incarceration has focused overwhelmingly on imprisonment (1, 2), yet the number who are admitted to local jails each year greatly exceeds the number admitted to prison. Unlike prisons, which incarcerate people convicted of felonies, jails detain defendants who are awaiting court action and those convicted for low-level offenses. In 2017, US prisons admitted about 600,000 people, but 10.6 million were sent to jail (3, 4).

Although duration is usually brief, jail incarceration has been found to adversely affect court outcomes, crime, and socioeconomic life. Researchers report that pretrial detention increases conviction rates, custodial sentences, court fees, and rearrest rates (5–8). Jail incarceration is also associated with reduced earnings and household income, increased risk of separation among parents, and reduced voter turnout (9–12). Because of the large number of jail admissions, researchers have argued that the aggregate effects of jail incarceration on life chances may also be proportionately large. As Turney and Conner wrote in their review, “understanding jail incarceration has important implications for understanding how the criminal justice system creates, maintains, and exacerbates social inequality” (13).

Current understanding of the jail has been shaped by research from the 1970s and 1980s. Jail incarceration focused on very poor urban residents who were often contending with homelessness and drug addiction. The jail was described managing “the rabble,” incarcerating “petty hustlers,” “derelicts,” “junkies,” and others that were distinguished not by their threat to public safety but their offensiveness to conventional norms and institutions (14, 15).

Contemporary studies suggest that the jail continues to incarcerate those facing persistent homelessness, untreated addiction, and mental illness. Consistent with the cooccurrence of poverty, homelessness, and poor health, poor prime-age adults in the United States grapple with reduced income support for long-term unemployment (16), rising healthcare costs and underinsurance (17), and a shrinking supply of affordable housing (18). Recent policing studies report on the use of arrest to keep the transient poor away from centers of tourism and business in a contemporary form of “rabble management” (19, 20). Research on criminal justice, healthcare, and homelessness points to the same group of individuals who simultaneously account for a large proportion of jail admissions, emergency rooms visits, and shelter stays (21, 22). Poor city residents with overlapping and chronic problems with health and housing have been called “frequent flyers” by criminal justice officials because of their high rate of repeated incarceration (23).

While the contemporary jail incarcerates a highly disadvantaged segment of the poor, recent research also describes an era of mass criminalization in which police, courts, and penal institutions exert a pervasive influence in low-income communities of color. From the 1980s, urban policing intensified enforcement against low-level offenses. Commonly described as order-maintenance, broken-windows, or quality-of-life policing, proactive efforts at enforcement against low-level offenses was often framed as a preventive strategy against serious crime. In New York City, for example, broken-windows policing was embraced by the police department in the 1990s and 2000s (24). Aggressive enforcement against low-level offenses evolved into “stop-and-frisk” policing that “blanket[ed] areas within a city with pedestrian stops” (25). Stop and frisk in New York was “concentrated in minority neighborhoods and conflated with poverty and other signs of socioeconomic disadvantage” (26) and was “tied more closely to demographic and socioeconomic conditions than crime” (27). The tactic generated millions of police stops in New York, disproportionately of Black and Latino men (28, 29). Similar rates of pedestrian stops were also reported in Los Angeles and Philadelphia, forming part of a broad trend to proactive policing that “diffused across the landscape of American policing” (25).

Intensified policing through order-maintenance enforcement filtered through to the courts. Most police stops in New York did not result in an arrest, yet the growth in stops was so large that the misdemeanor arrest rate also increased in the early 2000s (30). Among Black men aged 16 to 24 y, misdemeanor arrest rates peaked in 2011, the peak year for police stops. Among Black men in New York City, born 1976 to 1985, around a quarter are estimated to have been convicted of a misdemeanor offense by age 44 y (31).

In contrast to rabble management that focused on a small, highly disadvantaged segment of the population, jail incarceration under mass criminalization is a by-product of widespread arrests for drug and public-order offenses concentrated in low-income communities of color. Mass criminalization encompasses broken-windows policing (26, 32) and the “managerial justice” (33) of the misdemeanor courts that reserves wide discretion for police, prosecutors, and judges (34).

High rates of arrest and misdemeanor criminal processing have likely created a high risk of jail incarceration that is concentrated among Black and Latino men in low-income communities. Given the racial disparities in order-maintenance policing, we also expect high rates of repeated incarceration among Black and Latino men.

We assess the extent of mass criminalization by estimating cumulative risks of jail incarceration in New York City, where the police department pioneered the broken-windows policing that generated large numbers of misdemeanor arrests through the 1990s and 2000s (35). The focus on the cumulative risk of jail incarceration is motivated in part by research on the effects of jail and partly by the population dynamics of incarceration, in which the rate of jail detention is low compared to imprisonment but the admission rate is an order of magnitude higher.

Analyzing data on jail admissions from 2008 to 2017, we develop estimates of the cumulative risk of jail incarceration among New Yorkers by age 38 y. The analysis includes estimates for men and women separately, and for Blacks, Latinos, and Whites. We also estimate risks of incarceration in poor and nonpoor neighborhoods and the prevalence of repeated incarceration.

## Jail in New York City

Jail incarceration in New York City is supplied by five county jurisdictions, but defendants are detained in a single municipal system. Most people who are detained are held at the large Rikers Island jail complex. About three-quarters of those incarcerated on Rikers Island are awaiting court action on criminal cases, with the remainder sentenced up to a year on subfelony cases.

Reports of disorder and brutality at Rikers were widespread during the period of our analysis (36, 37). Popular pressure to close the jail escalated following reports of a suicide of a young man named Kalief Browder who had been held for 3 y, 17 mo in solitary confinement while awaiting trial (38). The chief medical officer of the jail described a culture of violence in which detainees were regularly beaten by correctional officers, yielding large numbers of head injuries, facial fractures, and lacerations requiring sutures (39). Solitary confinement was widely used at Rikers Island. About 7.5% of the population were held in punitive segregation on an average day in 2013, with an additional number diagnosed with mental illness in a facility dedicated to “restricted housing units” (40).

**Fig. 1.**
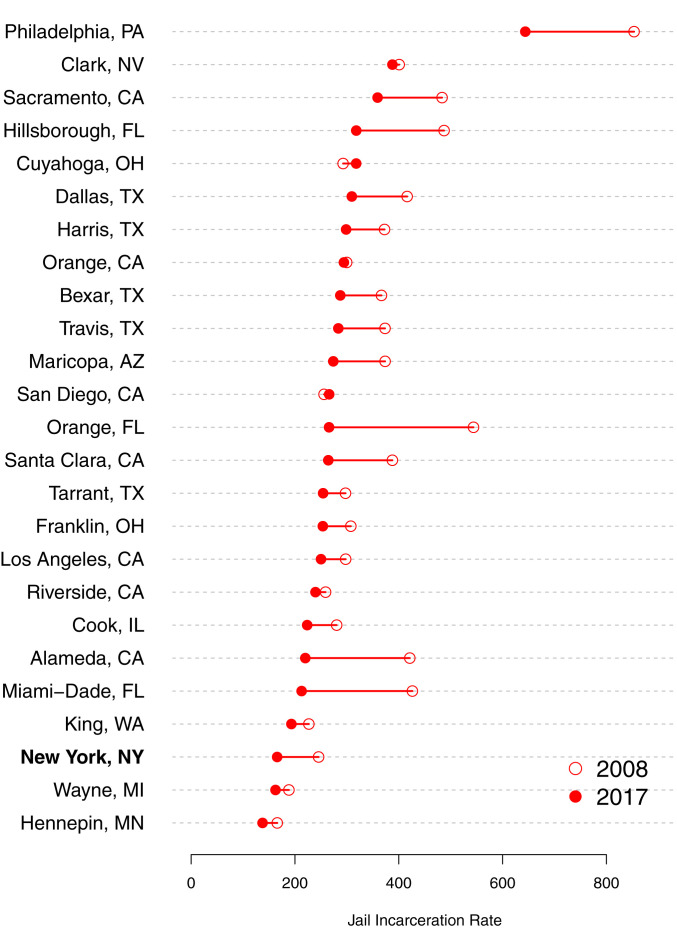
Jail incarceration rates in the 25 largest US counties, 2008 and 2017. New York, NY includes all five boroughs that make up New York City. The incarceration rate is the average daily jail population per 100,000 of the county population aged 15 to 64 y (42).

The problems of violence and mismanagement ultimately forced the New York mayor’s office to announce plans in 2017 to close the jail (41). The population had dropped significantly since its peak of more than 21,674 in 1991. In the period of the current analysis, from 2008 to 2017, the jail population declined from 13,849 to 9,500 (42). Fig. 1 compares the scale of jail incarceration New York City to other large urban counties in 2008 and 2017. Although New York’s jail system was historically the largest in the country, the jail incarceration rate by 2017 was the third-lowest.

New York City thus represents a strategically important site for studying jail incarceration. The city pioneered broken-windows policing and the experience of jail incarceration was unusually harsh because of violent conditions at Rikers Island. Still, with one of the lowest jail incarceration rates in the country, New York offers a conservative test of the extent of mass criminalization through the jail system.

The current analysis examines a 10-y administrative data file that records the dates of all jail admissions and releases, criminal charges, and demographic information. We analyze only admissions for New York City residents identified by their zip code of residence. Nearly 600,000 jail admissions were recorded by New Yorkers from 2008 to 2017 (Table 1). Similar to the national prison population, men account for about 90% of admissions to jail. Blacks and Latinos make up 52% of the New York City population (43) but about 90% of jail admissions. Unlike prisons, jails detain many people charged with minor crimes. About 56% of New York City jail admissions include those incarcerated for misdemeanors, outstanding warrants, or violations of parole. Violent felonies account for just 15% of all jail admissions. Jail stays tend to be short relative to prison incarceration. Whereas the median length of stay in state prison is 15 mo (44), the median period of detention in New York City is 11 d.

**Table 1.** Descriptive statistics for New York City jail admissions, by sex, race/ethnicity, and charge, 2008 to 2017

	Count of total admissions	Percentage of total admissions, %	Median age, y	Median prior admissions	Median days in jail, d
All admissions	598,648	100.0	32	4	11
Sex					
Male	535,394	89.4	32	5	11
Female	63,254	10.6	35	4	8
Race/ethnicity					
Black	341,476	57.0	32	5	11
Latino	192,813	32.2	31	4	11
White	47,191	7.9	36	3	11
Other	17,118	2.9	30	2	7
Top charge at admission					
Violent felony	88,589	14.8	25	2	19
Property felony	37,542	6.3	30	4	29
Drug felony	76,398	12.8	35	5	16
Other felony	58,289	9.8	29	3	14
Drug misdemeanor	59,686	10.0	40	9	6
Other misdemeanor	178,680	29.9	34	6	6
Warrants/other*	97,650	16.4	32	4	15

* Warrants/other includes jail admissions for outstanding warrants, parole violations, and vehicular summonses.

## Materials and Methods

We calculate the cumulative risks of jail incarceration adapting methods for the cumulative risks of imprisonment (45). Two key quantities are needed to calculate the cumulative risk of jail incarceration in New York City: 1) the number of people going to jail for the first time at each year of age, $J_{a}$, for ages a=16,17,…,38 y and 2) the population of New York City at risk for jail incarceration for the first time at each year of age, ${\tilde{P}}_{a}$. Before April 2017 in the state of New York, people who had been arrested could be admitted to jail through the adult criminal courts from the age of 16 y. The count of the number of people going to jail for the first time can be directly observed in the administrative data. For each person admitted to jail from 2008 to 2017, the data also include the total prior jail admissions from 1995 to 2007. With data on prior jail admissions, we can identify all first-time jail admissions in the period 2008 to 2017 for birth cohorts from 1979 to 2001. Those born in 1979 were 16 y of age in 1995 when jail admissions were first recorded and aged 38 y by 2017 when the observation period ends. Note that these data yield conservative estimates of total jail incarceration among New Yorkers, because only New York City jail admissions are counted.

Calculating cumulative risks up to age 38 y averages over all of the birth cohorts available in the data, yielding synthetic cohort estimates. At young ages, the age-specific incarceration risk is estimated from cohorts representing the whole observation period; only older cohorts are used at older ages. Synthetic cohort estimates of cumulative risks can be interpreted as the average risk across birth cohorts, in this case born 1979 to 2001. The estimates accurately measure the cumulative risk for any specific cohort, assuming that the underlying risk of incarceration is unchanging. In reality, the New York jail population is declining over the observation period. Below we also study how the decrease in the jail incarceration rate affects the cumulative risk of incarceration.

The population at risk for first-time jail incarceration is not observed directly, but it can be estimated with US census data and administrative jail data. The population of New Yorkers who have just turned 16 y of age in a given year, $P_{16}$, can be estimated with the population count of 16-y-olds from the 2010 census. The population at risk for first-time jail incarceration who are just turning 17 y of age, ${\tilde{P}}_{17}$, equals $P_{17}$ minus all those that went to jail at age 16 y, plus an adjustment for mortality and migration. More generally, at age a, the population at risk for first-time jail incarceration is

$${\tilde{P}}_{a}=P_{a}-J_{a-1}^{*},$$

where $J_{a}^{*}$ is the number of people who have ever been jailed in New York who have survived to age a. In the case of this local jurisdiction, survival includes those who have been jailed minus those who have left the city either through death or migration. We adjust for mortality among those who are jailed using a series of age-, race-, and sex-specific death rates published in New York City population projections (46). To adjust for out-migration, we estimate age-specific migration rates using New York data from pooled years of the American Communities Survey, 2005 to 2010 (47).

Research on urban out-migration among the formerly incarcerated and other disadvantaged groups indicates relatively low spatial mobility (48). The general population migration rates used here likely overestimate the spatial mobility of jail detainees, leading to underestimates of cumulative risks of incarceration. Mortality of the formerly incarcerated, on the other hand, tends to be higher than in the general population leading to overestimates of cumulative risks (49). Because the number of deaths is small compared to the number of jail admissions, estimates of cumulative risks do not depend strongly on the assumed death rate. Doubling the assumed mortality rate, for example, does not alter the main substantive findings (*SI Appendix*).

With estimates of first-time jail incarceration and the population at risk we can calculate the age-specific risk of jail among those who have never been incarcerated,

$$j_{a}=J_{a}/{\tilde{P}}_{a}.$$

Age-specific risks of first-time jail incarceration, $j_{a}$, can be used to expose a hypothetical population, yielding estimates of the cumulative risk of jail incarceration by age 38 y (see *SI Appendix* for details).

## Results

Estimates of the cumulative risks of jail incarceration are reported in Table 2. Among all male New Yorkers, over 13% are estimated to have been jailed by age 38 y. Consistent with mass criminalization, jail incarceration is extensive for minority men. Estimates indicate 27% of Black men and 16% of Latino men and have been jailed at least one time by age 38 y compared to only 3% of White men. Results for women also show high levels of racial disparity. About 5% of Black women in New York have been jailed compared to 2% of Latino women and fewer than 1% of White women.

**Table 2.** Cumulative risks of jail incarceration by age 38 y, by race/ethnicity, sex, and neighborhood poverty, New York City, 2008–2017

	All New York	Poor ZIP codes	Nonpoor ZIP codes	Poor/nonpoor ratio
Men				
All men	0.134	0.200	0.102	1.964
White	0.034	0.035	0.034	1.029
Black	0.268	0.330	0.223	1.484
Latino	0.162	0.191	0.137	1.393
Other	0.057	0.084	0.052	1.617
Women				
All women	0.022	0.034	0.016	2.165
White	0.007	0.011	0.006	1.728
Black	0.049	0.062	0.040	1.561
Latino	0.023	0.027	0.018	1.489
Other	0.006	0.010	0.005	1.945

Table 2 also reports cumulative risks for those living in poor and nonpoor neighborhoods. Poor neighborhoods are defined as those ZIP codes in the top third of poverty rates across New York. Our data only allow spatial disaggregation to the ZIP code level, likely underestimating differences by poverty status compared to more disaggregated measures such as census tracts.

Cumulative risks of jail incarceration for minority men vary substantially by ZIP code poverty rates. For Black men living in poor ZIP codes, we estimate that 33% have been jailed at least once by age 38 y, compared to 22% of those living in nonpoor neighborhoods. Among Latino men in poor neighborhoods, the cumulative risk of jail is 19%, more than five percentage points higher than in nonpoor neighborhoods. For White men, the risk of jail admission does not vary by neighborhood poverty status.

Cumulative risks of incarceration for women are higher in poor ZIP codes across all race and ethnic groups. Although their incarceration risk is very low, White women in poor ZIP codes face the highest relative risk compared to those in nonpoor ZIP codes. Relative disparities are smaller for minority women, but the jail incarceration risk is still about 50% higher for Black and Latino women who live in high-poverty communities.

**Fig. 2.**
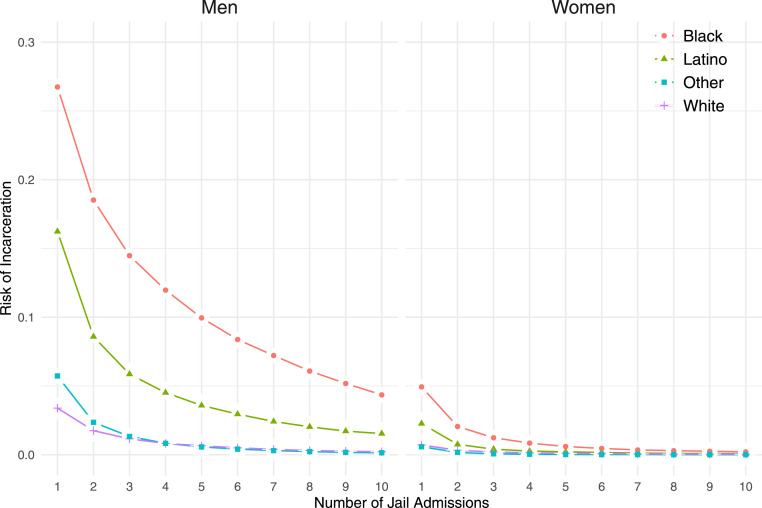
Cumulative risks of multiple jail admissions by age 38 y, by sex and race/ethnicity, New York City, 2008–2017

Because jails detain those arrested for low-level offenses for short periods of time, repeated incarceration is more common for jail than prison. Estimates of the cumulative risk of multiple jail admissions by race and ethnicity indicate that a relatively large proportion of Black and Latino men experience repeated jail incarceration (Fig. 2). Around 10% of Black men in New York and 4% of Latino men have been jailed five or more times by age 38 y. Around 2 to 4.5% of all Latino or Black men fit the pattern of constantly churning through the jail, accumulating 10 or more jail admissions by age 38 y. Black–White racial disparity increases with the number of jail incarcerations among men. Whereas Black men are 7.8 times more likely to be incarcerated at least once compared to White men, they are 20 times more likely to experience 10 or more jail admissions. By contrast, repeated incarceration is very uncommon among women. There are virtually no women, of any race or ethnicity, who have been incarcerated 10 or more times by age 38 y.

Finally, the synthetic cohort calculations most accurately describe the experience of specific birth cohorts if the age-specific risks are stable over time. The jail population is declining in the period of this analysis, from 2008 to 2017. In 2008, first-time jail admissions can only be identified for those aged 16 to 29 y, so we compare the cumulative risk of jail incarceration to age 29 y for each year from 2008 to 2017. Annual estimates of cumulative risks suggest how the shrinking of the jail population is affecting lifetime exposure to incarceration. Estimates for 2008 average over birth cohorts born between 1979 and 1992 and can be interpreted as the cumulative risks we would observe if jail admissions had remained constant at the 2008 level. Estimates for 2017 average over the 1988-to-2001 birth cohorts and reflect cumulative risks at the 2017 level of jail admission.

**Fig. 3.**
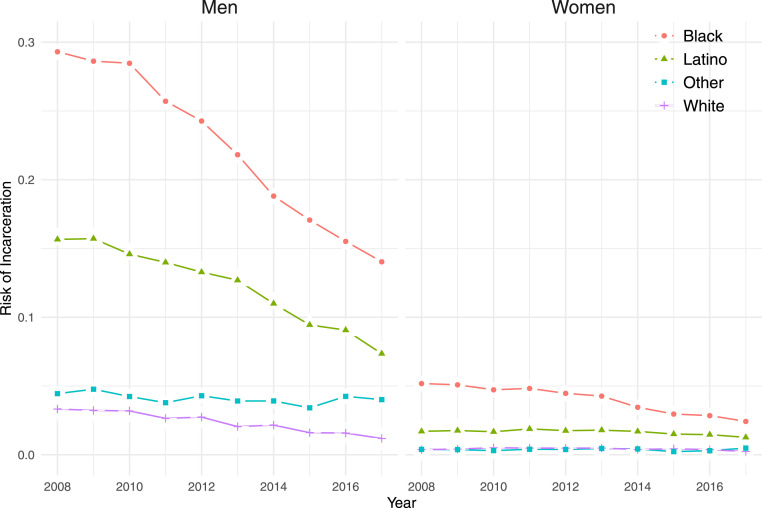
Changing cumulative risks of jail incarceration by age 29 y with declining jail population, by sex and race/ethnicity, New York City, 2008–2017

Cumulative risks of jail incarceration fall with the declining jail population for all racial and ethnic groups (Fig. 3). In 2008, when the jail population exceeded 13,000, nearly 30% of Black men and over 15% of Latino men could expect to be jailed by age 29 y. In 2017, when the jail population had shrunk by about one-third, the cumulative risk had fallen to under 15% for Black men and to about 7% for Latino men. Black women also recorded a large decline in cumulative risk, from 5.2% to 2.4%. Still, the largest proportionate decline was registered by White men, from 3.3 to 1.2%. Thus, the minority–White disparity in incarceration measured by the risk ratio increased even as Black and Latino men recorded large percentage point reductions in jail prevalence.

## Discussion

Research on the socioeconomic dimensions of incarceration has focused on imprisonment. The jail, however, is a far more dynamic institution, incarcerating many more people although for only a short period. Incarceration is brief, but jail time is disruptive, being associated with reduced employment, increased risk of criminal conviction, and rearrest.

Analysis of an administrative data file with all jail admissions in New York City from 2008 to 2017 supports three main empirical findings. First, in New York, where the jail incarceration rate is very low, there is great exposure to jail among Black and Latino men. Over a quarter of all Black men and a sixth of all Latino men have been incarcerated by age 38 y. Black–White racial disparity is particularly large. Black men’s relative risk of incarceration is 8 to 20 times higher than for White men, with the relative risk rising with the number of incarcerations. Reflecting the large racial disparity, Black women face higher risks of jail incarceration than White men.

Second, jail incarceration is spatially concentrated in high-poverty neighborhoods. For Black and Latino New Yorkers, the cumulative risks of going to jail were about 50% higher if they lived in a high-poverty neighborhood. The level and inequality in incarceration were particularly striking for Black men in poor neighborhoods. The high absolute and relative risks of Black men in high-poverty areas indicate that mass criminalization is concentrated at the intersection of race, poverty, and gender.

Third, reductions in the jail population from 2008 to 2017 have been associated with large absolute reductions in the cumulative risks of jail incarceration for Blacks and Latinos. In New York, the decline in the jail population has accompanied a significant decline in crime but also a change in policing policy that has reduced the number of misdemeanor arrests, particularly for drug crimes (30). The shift away from quality-of-life policing has coincided with not just a reduction in the jail population but also the prevalence of jail incarceration over the life course for New York residents.

Compared to cumulative risks of imprisonment, the current estimates indicate the high prevalence of jail incarceration given the incarceration rate and greater racial disparity. The New York City jail incarceration rate is only about a quarter the national imprisonment rate (166 compared to 698 per 100,000 for the 15- to 64-y-old population in 2017; *SI Appendix*), but the prevalence of jail is similar to that for imprisonment at the national level (50). Racial disparities in cumulative risks of men’s prison incarceration at the national level for similar but not identical birth cohorts, born 1975 to 1979, are less than 5 to 1 (50), while men’s Black–White ratio for New York jail incarceration is nearly 8 to 1.

The current estimates could be extended in two further directions to shed light on a policy regime in which criminal justice institutions have a leading role in the urban governance of poor communities. First, with data available over a longer historical period, it would be possible to estimate cohort changes in exposure to jail (45, 51). Such an analysis would document how the passage through adulthood for Black men in particular was increasingly interrupted by incarceration. If the jail functioned less to manage the social problems of homelessness, substance use, and mental illness and more to detain those swept up by quality-of-life policing, lifetime prevalence may have grown faster than the jail incarceration rate. Second, the jail incarceration rate in New York is well below the national average, and figures from other jurisdictions would help detail the role of jail in the lives of Black and Latino men nationwide. In cities where jail incarceration rates are twice as high, would the cumulative risks of jail be twice as high too? This is an empirical question that depends in part on the pattern of repeated incarceration and the length of stay in jail. If these other parameters were equal to New York’s, localities with double the incarceration rate would likely be jailing most Black men at least once by the time they reached midlife.

The contours of jail incarceration observed in New York City follow the pattern of mass criminalization where large numbers of Black and Latino men are subject to penal control, in most cases for low-level offenses. Jail incarceration is particularly extensive in poor neighborhoods. Just as repeated police contact has been documented under quality-of-life policing, repeated jail incarceration is also widely observed among Black men. The results suggest that the jail has greatly disrupted life for disadvantaged New Yorkers, but the scale of incarceration also shows historical variability. Reducing the jail population by a third over a decade changed the pathway through adulthood in communities of color as the prevalence of jail incarceration was halved for Black and Latino men.

## Supplementary Material

Supplementary File

## Data Availability

Data cannot be shared. The data have been provided by the New York City Department of Correction and are subject to state law that withholds the public availability of criminal records where a defendant receives an acquittal, dismissal, or other good result. About half the incarcerations in the analysis include sealed cases. The data were obtained under a Memorandum of Understanding with the Department of Correction that prevents the disclosure of identifying or deidentified data. *SI Appendix* includes the details of the current data request, and replication data could be obtained by filing a similar request.

## References

[1] S. Raphael, M. A. Stoll, Why Are So Many Americans in Prison? (Russell Sage Foundation, New York, 2013).

[2] J. Travis, B. Western, S. Redburn, The Growth of Incarceration in the United States: Exploring Causes and Consequences (National Academy Press, Washington, DC, 2014).

[3] Z. Zeng, “Jail inmates in 2017” (No. NCJ 251774, Bureau of Justice Statistics, Washington, DC, 2019).

[4] J. Bronson, E. A. Carson, “Prisoners in 2017” (No. NCJ 252156, Bureau of Justice Statistics, Washington DC, 2019).

[5] A. Gupta, C. Hansman, E. Frenchman, The heavy costs of high bail: Evidence from judge randomization. J. Leg. Stud. 45, 471–505 (2016).

[6] E. Leslie, N. G. Pope, The unintended impact of pretrial detention on case outcomes: Evidence from New York city arraignments. J. Law Econ. 60, 529–557 (2017).

[7] P. Heaton, S. Mayson, M. Stevenson, The downstream consequences of misdemeanor pretrial detention. Stanford Law Rev. 69, 711–794 (2017).

[8] M. T. Stevenson, Distortion of justice: How the inability to pay bail affects case outcomes. J. Law Econ. Organ. 34, 511–542 (2018).

[9] J. Grogger, The effect of arrests on the employment and earnings of young men. Q. J. Econ. 110, 51–71 (1995).

[10] W. Dobbie, J. Goldin, C. Yang, The effects of pre-trial detention on conviction, future crime, and employment: Evidence from randomly assigned judges. Am. Econ. Rev. 108 (2), 201–240 (2018).

[11] C. Wildeman, K. Turney, Y. Yi, Paternal incarceration and family functioning: Variation across federal, state, and local facilities. Ann. Am. Acad. Polit. Soc. Sci. 665, 80–97 (2016).

[12] A. White, Misdemeanor disenfranchisement? The demobilizing effects of brief jail spells on potential voters. Am. Polit. Sci. Rev. 113, 311–324 (2019).

[13] K. Turney, E. Conner, Jail incarceration: A common and consequential form of criminal justice contact. Annu. Rev. Criminol. 2, 265–290 (2019).

[14] J. Irwin, The Jail: Managing the Underclass in American Society (University of California Press, Berkeley, 1985).

[15] C. E. Frazier, E. W. Bock, J. C. Henretta, Pretrial release and bail decisions: The effects of legal, community, and personal variables. Criminology 18, 162–181 (1980).

[16] R. Haveman, R. Blank, R. Moffitt, T. Smeeding, G. Wallace, The war on poverty: Measurement, trends, and policy. J. Pol. Anal. Manag. 34, 593–638 (2015). 10.1002/pam.21846 PMC4822720 27064413

[17] L. Hawks, Trends in unmet need for physician and preventive services in the United States, 1998-2017. JAMA Int. Med. 180, 439–448 (2020). 10.1001/jamainternmed.2019.6538 PMC6990729 31985751

[18] R. Cox, B. Henwood, S. Rodnyansky, E. Rice, S. Wenzel, Road map to a unified measure of housing insecurity. Cityscape 21, 93–127 (2019).

[19] K. Beckett, S. Herbert, Banished: The New Social Control in Urban America (Oxford University Press, Oxford, 2010).

[20] F. Stuart, Down and Out and Under Arrest: Policing and Everyday Life in Skid Row (University of Chicago Press, 2016).

[21] S. Metraux, D. P. Culhane, Recent incarceration history among a sheltered homeless population. Crime Delinquen. 52, 504–517 (2006).

[22] R. MacDonald, The Rikers Island hot spotters: Defining the needs of the most frequently incarcerated. Am. J. Pub. Health 105, 2262–2268 (2015). 26378829 10.2105/AJPH.2015.302785 PMC4605192

[23] M. C. Ford, Frequent fliers: The high demand user in local corrections. Cal. J. Health Promo. 3, 61–71 (2005).

[24] W. J. Bratton, “Broken windows and quality-of-life policing in New York City” (New York Police Department, New York, NY, 2015).

[25] D. Weisburd, M. K. Majmundar, Proactive Policing: Effects on Crime and Communities (National Academies Press, Washington, DC, 2018).

[26] J. Fagan, G. Davies, Street stops and broken windows: Terry, race, and disorder in New York City. Fordham Urban Law J. 28, 457–504 (2000).

[27] J. Fagan, A. Geller, G. Davies, V. West, “Street stops and broken windows revisited: The demography and logic of proactive policing in a safe and changing city” in Race, Ethnicity, and Policing: New and Essential Readings, S. K. Rice, M. D. White, Eds. (New York University Press, 2010), pp. 309–348.

[28] A. Gelman, J. Fagan, A. Kiss, An analysis of the New York City police department’s ‘stop-and-frisk’ policy in the context of claims of racial bias. J. Am. Stat. Assoc. 102, 813–823 (2007).

[29] S. Goel, J. M. Rao, R. Shroff, Precinct or prejudice? Understanding racial disparities in New York city’s stop and frisk policy. Ann. Appl. Stat. 10, 365–394 (2016).

[30] M. Patten, “Trends in Misdemeanor Arrests in New York, 1980 to 2017” (John Jay College of Criminal Justice, New York, 2018).

[31] P. Hepburn, I. Kohler-Hausmann, A. Z. Medina, Cumulative risks of multiple criminal justice outcomes in New York city. Demography 56, 1–11 (2019). 31041605 10.1007/s13524-019-00781-7

[32] B. E. Harcourt, Illusion of Order: The False Promise of Broken Windows Policing (Harvard University Press, Cambridge, MA, 2001).

[33] I. Kohler-Hausmann, Misdemeanorland: Criminal Courts and Social Control in an Age of Broken Windows Policing (Princeton University Press, Princeton, NJ, 2018).

[34] A. Natapoff, Punishment without Crime: How Our Massive Misdemeanor System Traps the Innocent and Makes America More Unequal (Basic Books, New York, NY, 2018).

[35] P. Chauhan, “Trends in custody: New York City Department of Correction, 2000-2015” (John Jay College of Criminal Justice, New York, 2017).

[36] P. Bharara, J. Samuels, J. Powell, E. Daughtry, “Cripa investigation of the New York City Department Of Correction jails on Rikers Island” (Report of the United States Attorney’s Office for the Southern District of New York, New York, 2014).

[37] S. J. Martin, “Seventh report of the Nunez Independent Monitor” (Report of the Court-Appointed Monitor mandated by the Consent Judgment in Nunez v. City of New York et al., New York, 2018).

[38] J. Gonnerman, Before the law, *New Yorker*, 29 September 2014.

[39] H. Venters, Life and Death in Rikers Island (Johns Hopkins University Press, Baltimore, MD, 2019).

[40] C. Haney, J. Weil, S. Bakhshay, T. Lockett, Examining jail isolation: What we don’t know can be profoundly harmful. Prison J. 96, 126–152 (2016).

[41] Mayor’s Office of Criminal Justice, “Smaller, safer, fairer: NYC’s plan to close Rikers Island” (Office of the Mayor, City of New York, New York, 2018).

[42] Vera Institute of Justice, “Incarceration trends dataset” (Version 2.2, Vera Institute of Justice, New York, 2020).

[43] Department of City Planning, “Population growth and race/Hispanic composition” (Department of City Planning, City of New York, New York, 2011).

[44] D. Kaeble, “Time served in state prison, 2016” (Bureau of Justice Statistics, Washington, DC, 2018).

[45] B. Pettit, B. Western, Mass imprisonment and the life course: Race and class inequality in US incarceration. Am. Socio. Rev. 69, 151–169 (2004).

[46] J. J. Salvo, A. P. Lobo, E. Maurer, “New York City population projections by age/sex & borough, 2010–2040” (Department of City Planning, City of New York, New York, 2013).

[47] S. Ruggles, Data from “American Community Survey 2006–2010”(IPUMS USA, Version 10). https://usa.ipums.org/usa/. Accessed 25 September 2020.

[48] D. S. Kirk, A natural experiment on residential change and recidivism: Lessons from Hurricane Katrina. Am. Socio. Rev. 74 (3), 484–505 (2009).

[49] D. L. Rosen, V. J. Schoenbach, D. A. Wohl, All-cause and cause-specific mortality among men released from state prison, 1980–2005. Am. J. Pub. Health 98, 2278–2284 (2008). 18923131 10.2105/AJPH.2007.121855 PMC2636544

[50] B. Western, B. Pettit, Incarceration and social inequality. Daedalus 139, 8–19 (2010). 21032946 10.1162/daed_a_00019

[51] C. Wildeman, Parental imprisonment, the prison boom, and the concentration of childhood disadvantage. Demography 46, 265–280 (2009). 21305393 10.1353/dem.0.0052 PMC2831279

