# Supplementary material for: The cumulative risk of jail incarceration
Source: Proc Natl Acad Sci U S A. 2021 Apr 12;118(16):e2023429118. doi: 10.1073/pnas.2023429118 (PMC8072250; doi:10.1073/pnas.2023429118)
Supplement: Supplementary File [file pnas.2023429118.sapp.pdf]

PNAS
www.pnas.org

Supplementary Information for

The Cumulative Risk of Jail Incarceration

Bruce Western, Jaclyn Davis, Flavien Ganter, and Natalie Smith

Bruce Western.
E-mail: bruce.western@columbia.edu

This PDF file includes:

Supplementary text
Fig. S1 (not allowed for Brief Reports)
Tables S1 to S3 (not allowed for Brief Reports)
SI References

Supporting Information Text

Jail incarceration rate data reported in Figure 1 are taken from the Vera Incarceration Trends Database, which also provides county-level counts for the population aged 15 to 64 (1). With these data, the 2017 New York City jail incarceration rate per 100,000 of residents aged 15 to 64 is 165.7. By comparison, the national prison population in 2017 is 1,489,189 (2). A U.S. national population of 213,264,023 aged 15 to 64 (3) yields a national prison incarceration rate of 698.3 per 100,000 as reported in the Discussion of the paper.

Data Availability. The administrative data file used for cumulative risk estimation includes all admissions to New York City Department of Corrections custody from 2008 to 2017. These data were obtained under a Memorandum of Understanding (MOU) with the Department of Corrections. The terms of the MOU prevent the disclosure of any identified or de-identified data. To replicate our data, researchers would request all admissions and discharges from January 2008 through December 2017 including a person identifier for each incarceration as well as information on age, gender, race and ethnicity, ZIP code, admission and discharge dates, number of prior admissions, borough of arraignment, charge and warrant information, conviction date, sentence date, sentence length, and discharge reason.

Data Design and Lifetable Calculations. These data include jail admissions from a wide range of birth cohorts, and data are also available for all prior jail admissions from 1995 to 2007. Complete adult jail admission histories can thus be constructed for those born from 1979 (who are 16 in 1995) to 2001 (who are 16 in 2017). The structure of the data is shown in Figure S1 that reports a Lexis diagram that illustrates the contribution of each birth cohort to the analysis. Calculating cumulative risks up to age 38 averages over all the birth cohorts available in the data. The Lexis diagram shows that at young ages, the age-specific incarceration risk is estimated from cohorts representing the whole observation period; only later years are used at older ages. Synthetic cohort estimates of cumulative risks can be interpreted as the average risk across birth cohorts, in this case born 1979 to 2001. The estimates accurately measure the cumulative risk for any specific cohort, assuming that the underlying risk of incarceration is unchanging. In reality, the New York jail population is declining over the observation period and the paper reports how the decrease in the jail incarceration rate affects the cumulative risk of incarceration.

The paper describes the calculation of the age-specific risk of first-time jail incarceration, j_a ($a = 16, 17, \ldots, 38$). Assuming that an initial hypothetical population at age $a = 16$ exposed to the age-specific risk is $l_{16} = 100,000$ (called the radix), then the number incarcerated is $I_{16} = j_{16}l_{16}$, and more generally, $I_a = j_a l_a$. The cumulative risk is the sum of first-time incarcerations over the total population,

$$\text{Cumulative Risk} = \sum_{a=16}^{38} I_a/l_{16}.$$

To illustrate the the lifetable calculations, Table S1 reports the basic quantities for the entire sample of New Yorkers at each year of age averaging over all birth cohorts in the jail data. The raw counts of first jail admissions in column 1 of Table S1, are taken just from those who provide New York City residential addresses, about 80 percent of all jail admissions. The age-specific risk of first incarceration is highest at age 18, but significant numbers of first-time jail admissions continue past age 30. In the New York City population as a whole, for a synthetic cohort born 1979 to 2001, the risk of jail incarceration by age 38 is nearly 8 percent.

Migration and Mortality Assumptions. At age a, the population at risk of first-time incarceration depends on the number ever incarcerated who have survived to age a. Survival includes those who have not migrated or died. At ages 16 to 38, in a large urban area like New York, migration rates greatly exceed mortality rates. We estimated out-migration with general population data for New York from the American Communities Survey (4). Research suggest that the poor and the formerly-incarcerated experience lower rates of spatial mobility than the general population (5–7). Our general population estimates thus tend to over-estimate migration among those who have been to jail, leading to under-estimates of the age-specific risks of first-time jail incarceration, and under-estimates of the cumulative risks. We simply observe that our estimates are thus likely to be conservative due to the effects of differential migration.

The cumulative risk estimates reported in the paper were based on general population age-, race- and sex-specific mortality rates for New York City. Unlike migration rates, there is evidence that mortality rates are significantly higher among formerly-incarcerated men and women (see (8) for New York City results and (9, 10)). Excess mortality among the formerly-incarcerated will tend reduce estimates of cumulative risk, as the surviving cohort of jail detainees will be smaller than assumed. We study the sensitivity of the reported estimates assuming that mortality rates for jail detainees are three times those observed in the general population. Table S2 compares estimates assuming three times the mortality to the reported estimates. For all groups, assuming much higher mortality changes the reported estimates by less than one percent.

Calculating Cumulative Risks by ZIP Code. Our analysis also considers inequalities in incarceration across geographic areas. Racial disparities in incarceration result from disproportionately committing the residents of disadvantaged neighborhoods to prison and jail. Because of the spatial concentration of crime and policing in poor neighborhoods, incarceration too is closely associated with neighborhood poverty (11). In the era of mass incarceration, Robert Sampson (12, 13) has argued that spatially concentrated incarceration, along with racial segregation, crime, and poverty, have become a tightly clustered set of social conditions that together form an ecology of neighborhood disadvantage. New York City's spatial concentration of

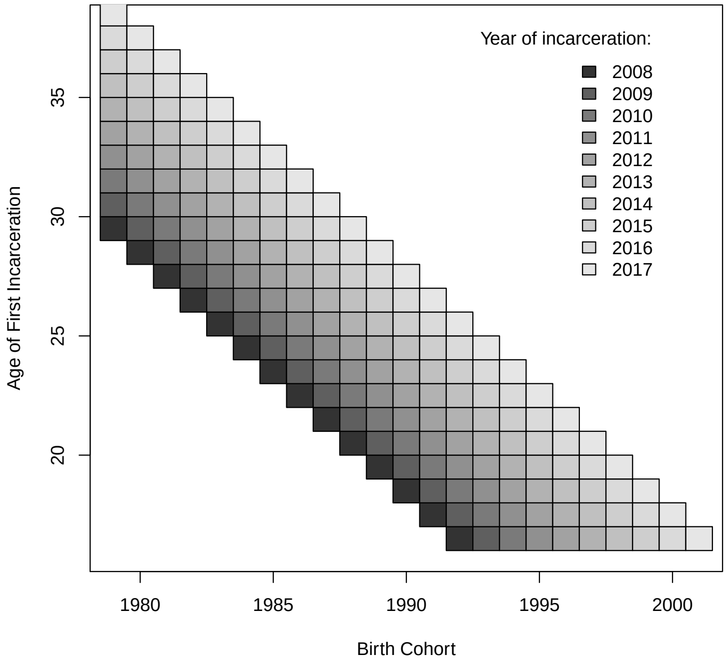

Fig. S1. Lexis diagram showing the contribution of each birth cohort by age and year of incarceration to synthetic cohort estimates of the cumulative risk of jail incarceration by age 38, New York City, 2008–2017.

Table S1. Lifetable calculations for risk of jail incarceration by age 38, New York City (2008–2017).

Age a (1)	Annual Count of First Incarceration J_a (2)	Age Specific Risk j_a (3)	Population at Risk l_a (4)	Cumulative Number Incarcerated $\sum I_a$ (5)	Cumulative Risk (6)
16	579	.006	100000	557	.006
17	714	.007	99443	1227	.012
18	758	.007	98773	1908	.019
19	737	.006	98092	2534	.025
20	703	.006	97466	3111	.031
21	646	.006	96889	3648	.036
22	600	.005	96352	4120	.041
23	546	.004	95880	4518	.045
24	526	.004	95482	4875	.049
25	473	.003	95125	5184	.052
26	424	.003	94816	5471	.055
27	406	.003	94529	5736	.057
28	377	.003	94264	5986	.060
29	345	.002	94014	6219	.062
30	322	.002	93781	6427	.064
31	286	.002	93573	6635	.066
32	266	.002	93365	6828	.068
33	249	.002	93172	7019	.070
34	230	.002	92981	7200	.072
35	205	.002	92800	7357	.074
36	175	.002	92643	7505	.075
37	164	.001	92495	7643	.076
38	144	.001	92237	7763	.078

Note: (1) Age in years. (2) Annual count of first incarceration is the annualized count of all New Yorkers entering jail for the first time at each age. (3) The age-specific risk is the number entering jail divided by the observed population of New Yorkers at risk of first incarceration, adjusting for mortality and migration. (4) The population at risk is a hypothetical population (called the radix at the baseline age) that have not yet been incarcerated. (5) The cumulative number incarcerated is the cumulative sum of the age-specific risk times the population at risk. (6) The cumulative risk is the cumulative number incarcerated divided by the radix.

Table S2. Sensitivity of cumulative risk to mortality rate assumptions. Cell entries are the ratio of alternative cumulative risk estimates based on three times the observed mortality to the cumulative risks reported in Table 2.

	All New York	Poor ZIPcodes	Nonpoor ZIPcodes
Men			
All men	.999	.999	1.000
White	1.000	1.000	1.000
Black	.997	.996	.997
Latino	.999	.999	.999
Other	1.000	1.000	1.000
Women			
All women	1.000	1.000	1.000
White	1.000	1.000	1.000
Black	1.000	1.000	1.000
Latino	1.000	1.000	1.000
Other	1.000	1.000	1.000

incarceration has been documented at least since the early 1990s, when prison reform advocates calculated that 75 percent of the state's prison population came from just seven neighborhoods in the city (14).

The jail administrative file includes a ZIP code of residence for each jail admission. The data are used to calculate first-time jail admissions, by age, from each of New York's 145 ZIP codes. Population information for each ZIP code is taken from the 2010 Census. Census poverty rates are reported for ZIP Code Tabulating Areas (ZCTAs) by mapping census blocks to ZIP code boundaries. High poverty ZIP codes were defined as ZCTAs in the top tercile by poverty rate.

To study robustness across other neighborhood characteristics, we also estimated cumulative risks by neighborhod crime rate. New York City provides exact addresses for all crimes reported to police. We assigned all reported violent crimes to ZIP codes, 2008–2017, to identify ZIP codes in the top tercile by crime rate. Once the high-crime ZIP codes were identified, census population data for ZCTAs were used to calculate cumulative risks. The pattern of results is similar to that reported for poverty (Table S3).

References

1. Vera Institute of Justice, *Incarceration Trends Dataset Version 2.2.* (Vera Institute of Justice, New York, NY), (2020).
2. EA Carson, Prisoners in 2017 (Bureau of Justice Statistics NCJ 253516) (2020).
3. U.S. Census Bureau, Population Division, Annual estimates of the resident population for selected age groups by sex for the united states: April 1, 2010 to july 1, 2019 (nc-est2019-agesex) (Downloaded from: https://www2.census.gov/programs-surveys/popest/technical-documentation/file-layouts/2010-2019/nc-est2019-agesex-res.csv) (2020).
4. S Ruggles, et al., American community survey 2006–2010 (datafile) (IPUMS USA Version 10) (2019).
5. DS Kirk, A natural experiment on residential change and recidivism: Lessons from hurricane katrina. *Am. Sociol. Rev.* **74**, 484–505 (2009).
6. P Sharkey, RJ Sampson, Destination effects: Residential mobility and trajectories of adolescent violence in a stratified metropolis. *Criminology* **48**, 639–81 (2010).
7. JT Simes, Place after prison: Neighborhood attainment and attachment during reentry. *J. Urban Aff.* **41**, 443–463 (2018).
8. S Lim, TG Harris, D Nash, MC Lennon, LE Thorpe, All-cause, drug-related, and hiv-related mortality risk by trajectories of jail incarceration and homelessness among adults in new york city. *Am. J. Epidemiol.* **181**, 261–270 (2015).
9. IA Binswanger, PJ Blatchford, SR Muller, MF Stern, Mortality after prison release: Opioid overdose and other causes of death, risk factors, and time trends from 1999 to 2009. *Annals Intern. Medicine* **159**, 592–600 (2013).
10. DL Rosen, VJ Schoenbach, DA Wohl, All-cause and cause-specific mortality among men released from state prison, 1980–2005. *Am. J. Public Heal.* **98**, 2278–2284 (2008).
11. JT Simes, Place and punishment: The spatial context of mass incarceration. *J. Quant. Criminol.* **34**, 515–533 (2018).
12. RJ Sampson, *Great American City: Chicago and the Enduring Neighborhood Effect.* (University of Chicago Press, Chicago, IL), (2012).
13. R Manduca, RJ Sampson, Punishing and toxic neighborhood environments independently predict the intergenerational social mobility of black and white children. *Proc. Natl. Acad. Sci.* **116**, 7772–7777 (2019).
14. J Gordon, Learned in the classroom, digested in the yard. *New Dir. for Adult Continuing Educ.*, 23–35 (2019).

Table S3. Cumulative risks of jail incarceration by crime rate of ZIP code, New York City, 2008–2017.

	High-Crime ZIP codes	Low-Crime ZIP codes	High/Low Crime Ratio
Men			
All men	.220	.097	2.265
White	.051	.032	1.609
Black	.311	.224	1.389
Latino	.197	.141	1.396
Other	.124	.048	2.564
Women			
All women	.039	.015	2.652
White	.019	.006	3.221
Black	.058	.040	1.448
Latino	.029	.018	1.574
Other	.014	.005	2.920
